# Validity of the Derby Scale of Intrinsic Capacity and its association with Pain: a usable measurement of intrinsic capacity based on the English Longitudinal Study of Ageing

**DOI:** 10.1007/s41999-026-01558-z

**Published:** 2026-07-23

**Authors:** Jemima T. Collins, Christopher Newby, Rowan H. Harwood, Adam L. Gordon

**Affiliations:** 1 Injury, Recovery and Inflammation Sciences, School of Medicine, University of Nottingham, Nottingham, NG7 2RD UK; 2 Department of Medicine for the Elderly, University Hospitals of Derby and Burton NHS Foundation Trust, Derby, UK; 3 Research Knowledge Exchange, School of Medicine, University of Nottingham, Nottingham, UK; 4 School of Health Sciences, University of Nottingham, Nottingham, NG7 2HA UK; 5 Academic Centre for Healthy Ageing, Barts Health NHS Trust, Whipps Cross Hospital, London, UK; 6 Wolfson Institute of Population Health, Queen Mary University of London, London, UK

**Keywords:** Intrinsic capacity, Pain, Validation

## Abstract

**Introduction:**

Intrinsic capacity (IC) is the composite of an individual’s physical and mental capacities which maintain functional ability. We aimed to develop and validate an IC scale that was ready for clinical use, and to explore the interaction between pain, IC, and functional outcomes.

**Methods:**

Confirmatory factor analysis was used to test the construct validity of the model drawing on items from the English Longitudinal Study of Ageing (ELSA). The final Derby Scale of Intrinsic Capacity (DSIC) included mobility (Short Physical Performance Battery), cognition (orientation and 10-word recall), vitality (body mass index), sensory (self-reported vision and hearing), and psychology (Satisfaction with Life). Logistic regression, repeated-measures ANOVA, and structural equation modeling were used to explore interactions between pain, IC, and outcomes.

**Results:**

2486 (wave 6) out of 4052 (wave 4) people aged ≥ 60 years had complete data for analysis. A higher (better) baseline DSIC was associated with decreased risk of basic and instrumental activities of daily living [OR 0.73, 95% CI 0.67–0.80], informal help [OR 0.69, 95% CI 0.62–0.78], formal help [OR 0.66, 95% CI 0.53–0.82], and falls [OR 0.90, 95% CI 0.83–0.97]. People with pain at baseline and follow-up had a lower (poorer) DSIC than those with no pain. The model we created accounted for 36% of variability of ADL difficulties. Pain reduced DSIC by 12% and in turn ADL difficulties by a further 12%.

**Conclusion:**

The DSIC is a validated, clinically user-ready tool for measuring IC in older people. Pain was associated with poorer IC and mediated the relationship between IC and ADL difficulties.

**Supplementary Information:**

The online version contains supplementary material available at https://doi.org/10.1007/s41999-026-01558-z.

## Key summary points

**Aim:**

This study developed and validated a clinically ready-to-use intrinsic capacity (IC) scale–the Derby Scale of Intrinsic Capacity (DSIC) and explored how pain, intrinsic capacity, and functional outcomes interact.

**Findings:**

A better baseline DSIC was associated with decreased risk of activities of daily living. Pain was associated with poorer IC and mediated the relationship between IC and ADL difficulties.

**Message:**

The DSIC is a validated tool to measure intrinsic capacity, enabling measurement of IC to facilitate targeted intervention.

**Supplementary Information:**

The online version contains supplementary material available at https://doi.org/10.1007/s41999-026-01558-z.

## Introduction

Between 2015 and 2050, the proportion of people over the age of 60 years is projected to rise from 12 to 22% [1]. Against this background, the World Health Organisation (WHO) has advocated for a change of focus from disease and morbidity to maintenance of functional ability. Functional ability, the health-related attributes which enable people to be and to do what they have reason to value, results from the interaction between an individual’s environment and their intrinsic capacity [2]. Intrinsic capacity (IC) is comprised of five domains—sensory (vision and hearing), cognition, vitality, locomotion, and psychological capacity [1]. Regular assessment of these domains over time enables early detection of declines in IC, highlighting needs for medical or rehabilitation care [3].

Chronic pain is both prevalent and debilitating. Pain is a multidimensional sensory and emotional experience [4]. Aspects or symptoms within each intrinsic capacity domain may be linked with pain, where the strongest associations are demonstrated in psychological, cognitive, and locomotion domains. These aspects include depression [5], non-restorative sleep [6], cognitive impairment [7, 8], and reduced gait speed [9]. Chronic pain may also contribute to the development of functional difficulties and disability [10–12].

Bellelli and colleagues explored the cross-sectional relationship between pain intensity and IC in a French cohort study comprising 971 people with an age range of 20–102. They found that people with higher pain intensities had lower intrinsic capacity and that this association was not moderated by inflammatory aging [13]. Yuan et al. explored pain and IC in a Chinese cohort and found older people with either single- or multi-site pain had lower IC compared to older people with no pain [14]. Although these studies helpfully report the association between pain and IC, they do not provide insights into how pain and IC interact over time, in a way that might inform development of interventions to manage pain and maintain IC.

Within the English Longitudinal Study of Ageing (ELSA), IC scores have validity both for underlying biological theory of capacity and for predicting difficulties with ADL, IADL, hospitalization, and mortality [15, 16]. Both these models generate scores based on mathematical calculations of how reliably the response to items reflects a true latent IC global factor or score, but neither is standardizable in its current form, nor clinically ready to use.

The five domains of the WHO IC concept were agreed by consensus [17]. In clinical practice, however, IC domain items and their combinations vary widely from measurements derived by an interviewer to self-reported questions. The primary aim of this study was to use existing variables within the ELSA dataset in order to build an IC scale with construct and predictive validity suitable for everyday clinical use, compared to an established model that is not clinically practical. A secondary aim was to explore the interaction between pain and IC longitudinally, and the effect of these interactions on functional outcomes.

## Methods

### Study design and population

The English Longitudinal Study of Ageing (ELSA) is an ongoing national cohort study following a sample of the population living in private accommodation in England [18]. Participants in ELSA provided written consent prior to the study, where health and care data were collected every 2 years and nurse visits at 4-year intervals. Ethical approval for Wave 4 was granted by the National Hospital for Neurology and Neurosurgery & Institute of Neurology Joint Research Ethics Committee (07/H0716/48) and for Wave 6 by the National Research Ethics Service Committee South Central-Berkshire (11/SC/0374). Wave 4 data were collected between 2008 and 2009 and Wave 6 data between 2012 and 2013. All data were made available through the UK Data Service.

### Sample

**Fig. 1:**
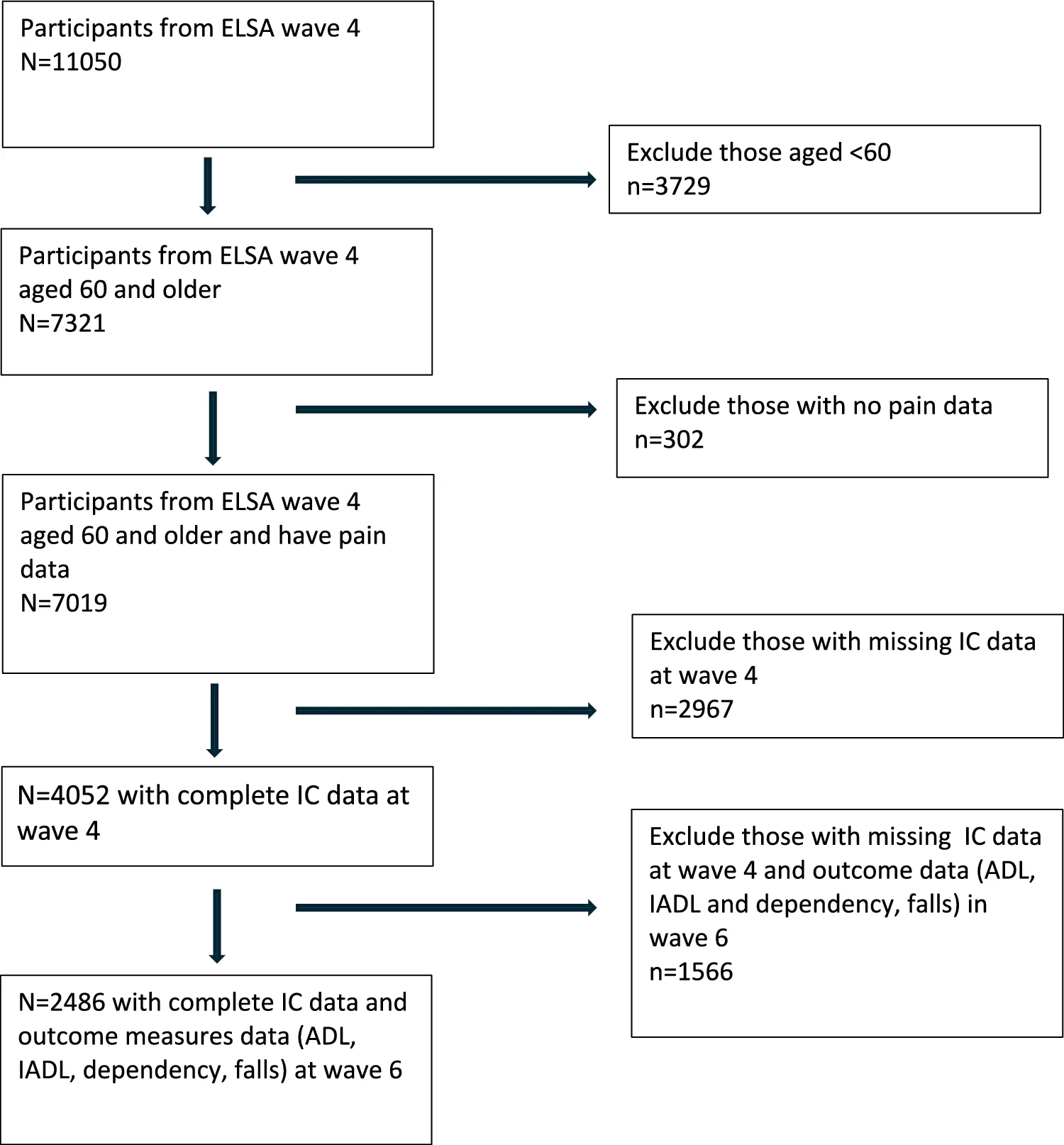
Flowchart of study participants from the English Longitudinal Study of Aging (ELSA) Wave 4 and Wave 6

For construct validity, we included participants 60 years old and above from wave 4 with no missing data in IC or pain items. For predictive validity, and to explore longitudinal interactions between IC and pain, we analyzed a subset of people who had complete IC, pain, and outcomes data for both waves 4 and 6 (Fig. 1).

### Intrinsic capacity (IC) measurement

IC was calculated based on the WHO five-domain concept of locomotion, vitality, senses, psychology, and cognition. The criteria for items to be included in the new scale were: (i) previous evidence confirming an association with domains of intrinsic capacity; (ii) high loading on confirmatory factor analysis (CFA); and (iii) exclusion of redundant items that load onto the same factor.

Each domain was scored between 0 and 2, with a resulting total score of 0–10 where 0 was the lowest (poorest) and 10 the highest (best) [19]. The scale was named the Derby Scale of Intrinsic Capacity (DSIC), which is detailed below (refer to Supplementary files for the full scoring system). This scale is intended to be free to use with acknowledgement but without further permission.

Locomotion was assessed by Short Physical Performance Battery (SPPB) [20]. This comprised interviewer-assessed timed tests of balance, sit-to-stand, and gait speed. We assigned scores of 0 for SPPB 0–6 (low), 1 for SPPB 7–9 (intermediate), and 2 for SPPB 10–12 (high) [21, 22].

Vitality was assessed by body mass index (BMI), which is weight in kilograms divided by height in meters squared. We assigned scores of 0 for BMI < 18.5 (underweight) and BMI ≥ 30 (obese), 1 for BMI 25–29.9 (overweight), and 2 for BMI 18.5–24.9 (healthy weight) for the vitality domain [23].

Senses were assessed by self-reported vision and hearing. Scores were assigned as 0 for poor vision and hearing, 0.5 as fair or good vision and hearing, and 1 as very good or excellent vision and hearing [19], with a total range of 0–2 with increments of 0.5.

Psychology was assessed by the Satisfaction with Life Scale (SWLS) [24], a five-item scale measuring an individual’s global evaluation of their life, with seven possible responses per item. We assigned scores of 0 for SWLS 0–19 (dissatisfied), 1 for SWLS 20–24 (neutral), and 2 for SWLS 25–35 (satisfied) [25].

Cognition was assessed by aspects of orientation (knowing the day of the week, day of the month, month, and year accurately) and 10-word immediate recall. Knowing all 4 orientation questions was good, and recalling 5 or more words was good [26]. We assigned scores of 0 for both aspects being poor, 1 to either but not both aspects being good, and 2 to both aspects being good.

### Pain assessment

Pain was evaluated in two ways. For predictive validity and evaluation of the interaction between pain and intrinsic capacity, the question “are you often troubled with pain?” has response options of yes or no. For structural equation modeling, pain intensity was coded as having no pain, mild pain, moderate pain, or severe pain.

### Outcome measures

Functional outcome measures were basic activities of daily living (ADL), instrumental activities of daily living (IADL), requirement for informal help and formal help, and falls. There were 6 basic ADL which recorded difficulties with dressing, walking across a room, transferring, bathing, eating, and using the toilet. There were 9 IADL which recorded difficulties with using a map, preparing a hot meal, shopping for groceries, making telephone calls, communication, recognizing when in physical danger, taking medication, doing work in the house, and managing money. These were recorded from wave 6.

### Covariates

Baseline covariates were chosen for factors that are potential confounders between intrinsic capacity and health outcomes in older people. They were age, sex, marital status, employment status, highest educational qualification, smoking, alcohol consumption, physical activity levels, and number of health conditions (Supplementary files).

### Statistical analysis

Statistical analysis was performed using SPSS version 31 [27] and the lavaan package in R. In order to generate the DSIC score, we first performed confirmatory factor analysis. The predetermined factor structure was based on the items used by Campbell et al. [16], which was replicated in this study. Construct validity of the DSIC was tested visually using a scatterplot comparing the DSIC and the factor scores, and Spearman’s rho correlation coefficient was used to check that items included in the final DSIC correlated well with the subfactor loading from Campbell et al. [16]. Predictive validity of the DSIC for each outcome measure was tested using binary logistic regression, adjusted for covariates. Exp(*B*) as odds ratios (OR) and 95% confidence intervals were reported.

Next, we explored the interaction between pain and DSIC. People were divided into four groups for comparison: those with pain in wave 4 and pain in wave 6 (pain–pain), those with pain in wave 4 and no pain in wave 6 (pain–no pain), those with no pain in wave 4 and pain in wave 6 (no pain–pain), and those with no pain in wave 4 and no pain in wave 6 (no pain–no pain). Repeated-measures ANOVA was used to test the relationship between the DSIC longitudinally, and present this graphically according to the four pain groups.

Finally, structural equation modeling (SEM) was employed to explore the extent to which pain mediated the effect of intrinsic capacity on ADL and IADL in people experiencing pain. This was presented graphically with standardized beta coefficients and in a table with unstandardized beta coefficients, 95% CI, and corresponding *P*-values.

## Results

A flowchart of participants included is presented in Fig. 1 for both waves 4 and 6. Out of 11,050 participants in wave 4, 4052 participants aged 60 years or more and had complete exposure variables of pain and IC. They were included in the baseline analysis. Of these, 2486 participants had complete intrinsic capacity and outcome measures data at follow-up (wave 6) and were included in the final analysis.

At baseline, the mean (SD) age of the sample was 69 (7.0) and 52% were female. The mean DSIC score for the group at baseline was 7.44 (SD 1.43) (Supplementary file) with 0% having the minimum score of 0 and 4% having the maximum score of 10. We present the covariates at baseline according to groups of people with pain and no pain (Supplementary file).

Confirmatory factor analysis revealed five factors with eigenvalues greater than 1. These accounted for 69% of total variance among intrinsic capacity indicators. Taking into account variables considered redundant for an abbreviated user-friendly scale, and according to the criteria set out in the intrinsic capacity measurement section, we incrementally removed self-reported lower limb and upper limb difficulty, handgrip strength, waist circumference, and the Cornell Score for Depression (CES-D). This was because they loaded highly on subfactors together with variables considered theoretically more valuable. The subfactor variables correlated well with the original scale (Spearman’s rho 0.56 for mobility, 0.90 for vitality, 0.75 for psychology, 0.78 for cognition, and 0.89 for sensory domains). Supplementary Fig. 1 shows the plot of the DSIC score against the original, with a *R*^2^ linear correlation coefficient value of 0.720 showing strong correlation and construct validity of the DSIC.

Binary logistic regression analyses revealed that the DSIC was predictive of important functional outcomes in an inverse relationship (Table 1). Each functional outcome measure was dichotomized into any difficulty in each area or no difficulty. Those with a higher DSIC score at baseline were 27–35% less likely to experience requirements for informal or formal help or difficulties with ADL or IADL (OR values of 0.65–0.73, all *P* < 0.001), and 10% less likely to have had a fall (OR 0.90, *P* < 0.01) 4 years later.

**Table 1:** Unadjusted and adjusted OR and significance values for baseline intrinsic capacity as a predictor of future health and care outcomes

	Unadjusted			Adjusted for age and sex			Adjusted for age, sex, comorbidities, marital status, employment, alcohol intake and physical activity and smoking		
	OR (95% CI)	SE	*P* value	OR (95% CI)	SE	*P* value	OR (95% CI)	SE	*P* value
Any ADL difficulty	0.58 (0.53, 0.64)	0.05	< 0.001	0.59 (0.54, 0.66)	0.052	< 0.001	0.65 (0.58, 0.73)	0.06	< 0.001
Any IADL difficulty	0.64 (0.58, 0.70)	0.05	< 0.001	0.65 (0.60, 0.72)	0.048	< 0.001	0.71 (0.64, 0.80)	0.06	< 0.001
Any ADL or IADL difficulty	0.66 (0.61, 0.71)	0.04	< 0.001	0.67 (0.62, 0.73)	0.039	< 0.001	0.73 (0.67, 0.80)	0.04	< 0.001
Falls	0.89 (0.83, 0.95)	0.03	< 0.001	0.89 (0.83, 0.95)	0.034	< 0.001	0.90 (0.83, 0.97)	0.04	0.006
Informal help	0.64 (0.58, 0.71)	0.05	< 0.001	0.66 (0.60, 0.73)	0.051	< 0.001	0.69 (0.62, 0.78)	0.06	< 0.001
Formal help	0.56 (0.47, 0.67)	0.09	< 0.001	0.61 (0.50, 0.73)	0.094	< 0.001	0.66 (0.53, 0.82)	0.11	< 0.001

*ADL* activities of daily living, *IADL* instrumental activities of daily living, *Exp(B)* exponential of beta, *SE* standard error

**Fig. 2:**
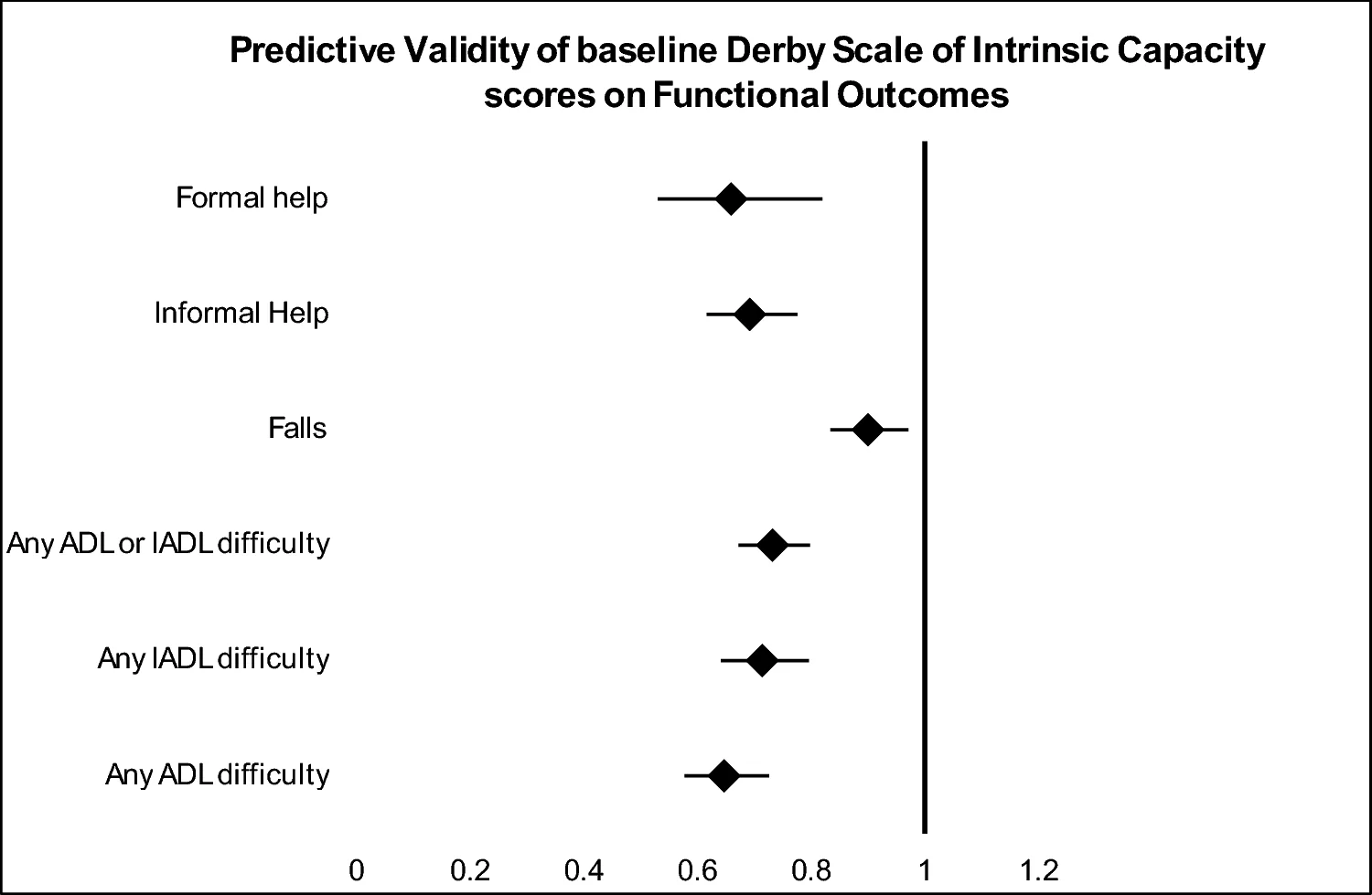
Forest plot of logistic regression analyses of baseline intrinsic capacity scores predicting future health and social care outcomes. Fully adjusted OR with 95% confidence interval values are presented

Figure 2 presents the future risk of having difficulties with ADL and IADL, requiring informal or formal help, and having one or more falls in the preceding year in a forest plot (Fig. 2).

**Fig. 3:**
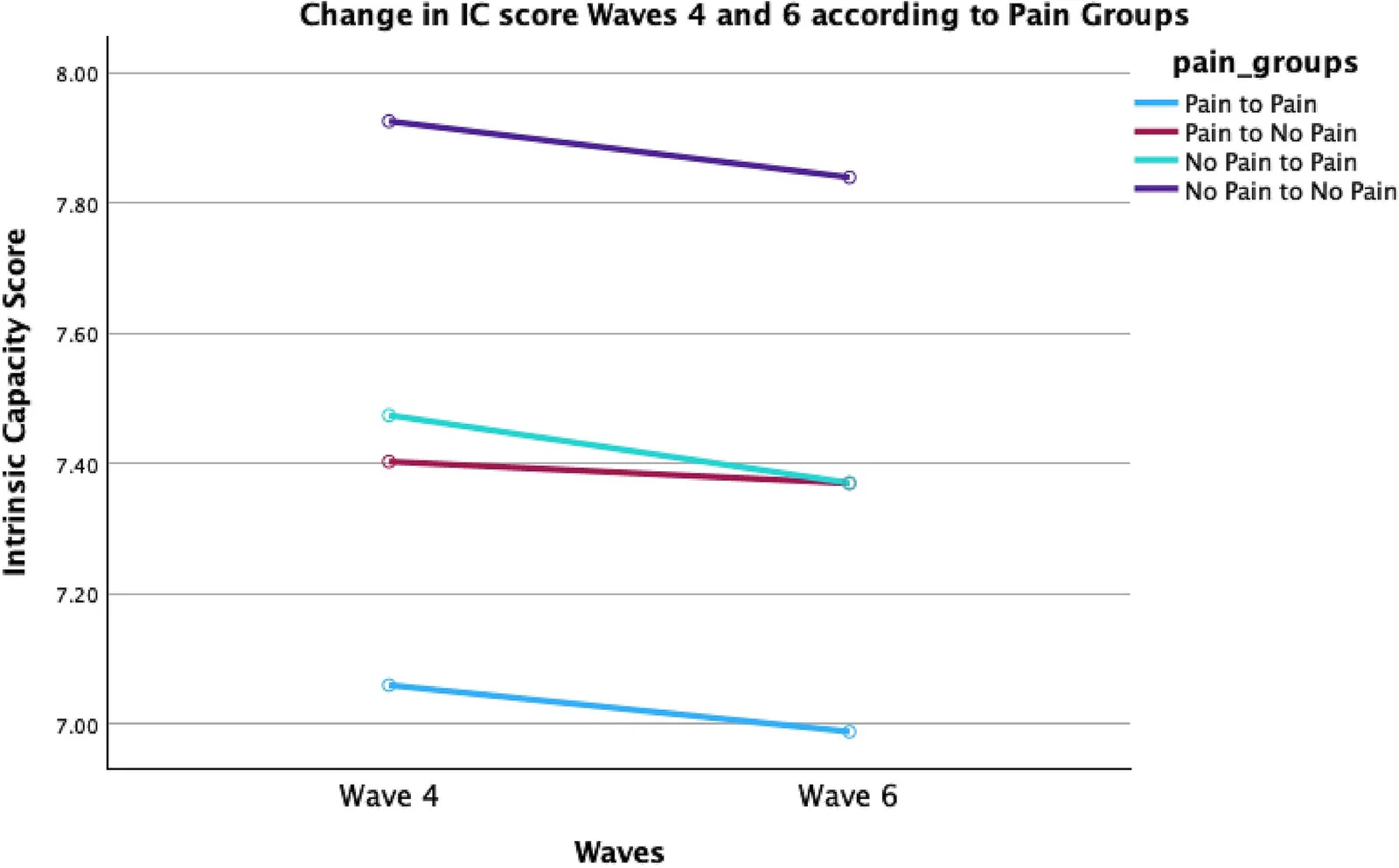
Repeated-measures ANOVA showing changes in intrinsic capacity scores longitudinally according to pain groups

Repeated-measures ANOVA (Fig. 3) revealed that participants in the pain–pain group were more likely to have a lower DSIC score at baseline and follow-up, compared to those in the other three groups. Participants with pain–no pain had an overall more maintained DSIC score compared to the other three groups.

Participants in the pain–pain group were more likely than the groups of pain–no pain, no pain–pain, and no pain–no pain to experience falls (34% vs 25%, 23% and 22%), difficulties in ADL or IADL (42% vs 20%, 23% and 9%), and require informal help (20% vs 9%, 10% and 4%) or formal help (5% vs 3%, 2% and 1%) (Supplementary files).

**Fig. 4:**
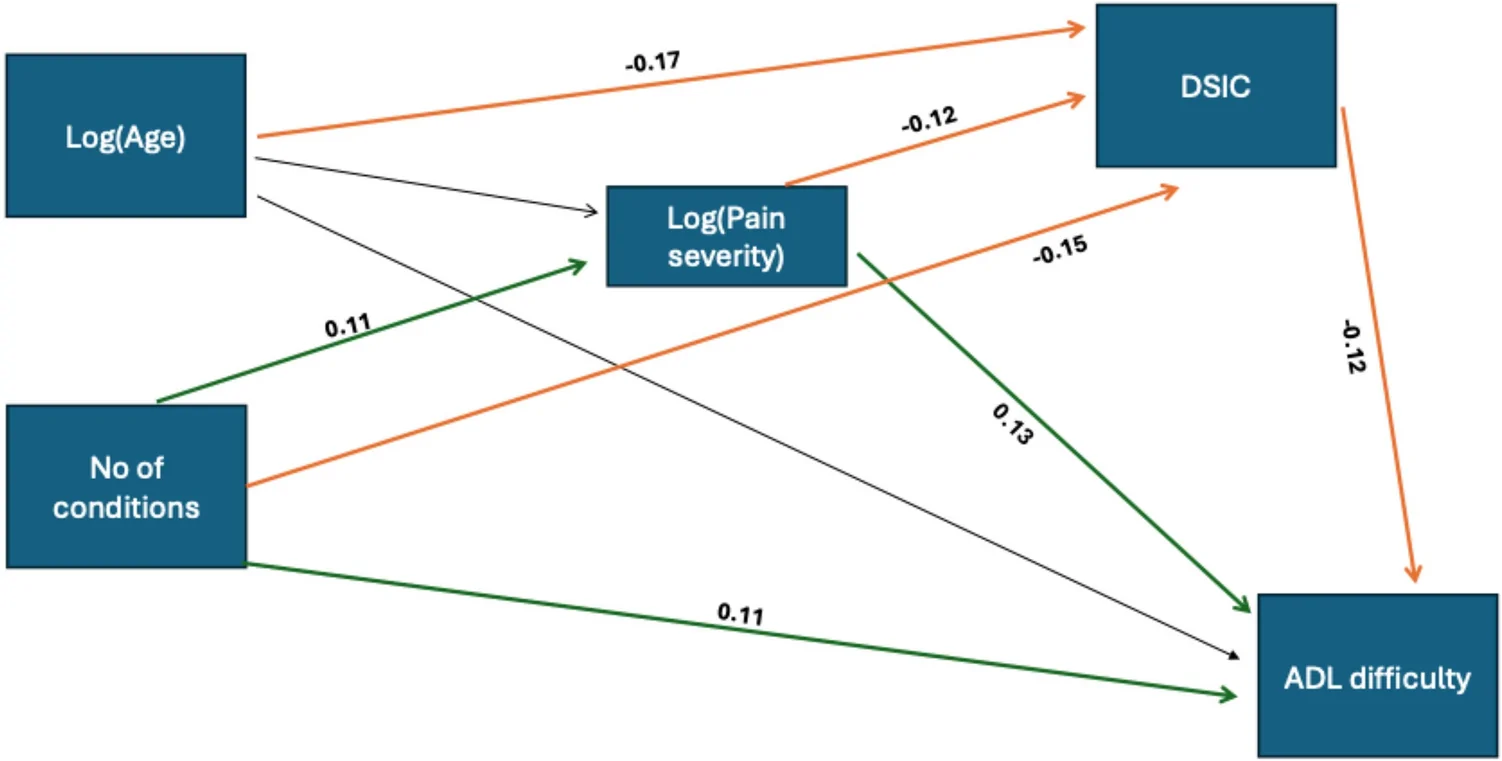
Structural equation modeling of the effects of age and number of comorbidities on Activities of Daily Living difficulties, moderated by pain and intrinsic capacity. Figures stated on arrows are Standardized Beta. Only significant Standardized Beta values are shown, all *P* < 0.01. Green arrows indicate a positive relationship, and orange arrows indicate an inverse relationship

The SEM model defined the number of ADL difficulties as a continuous construct. We found that ADL difficulties were significantly affected by number of comorbidities and pain in a direct relationship and DSIC score in an inverse relationship, but not by age directly. The models accounted for 36% of variability of ADL difficulties and 37% of IADL difficulties. As both these models were very similar, the ADL difficulties model is presented in Fig. 4 and the IADL model in Supplementary files. Pain increased the likelihood of ADL difficulties by 13% of the SD directly, but was also mediated by IC (reducing DSIC by 12% and in turn ADL difficulties by a further 12%) (Fig. 4 and Supplementary Table 4).

## Discussion

This study has generated an easily calculated and validated asset-based measurement scale—the Derby Scale of Intrinsic Capacity (DSIC). This scale will facilitate real-world scoring of intrinsic capacity. We also showed how using the scale in the context of pain could describe differential IC trajectories and demonstrated the mediating interaction of pain on IC and functional outcomes.

Our findings are consistent with the existing literature that IC is associated with wider health and functional outcomes. A meta-analysis with over 206,000 people showed that there was a longitudinal inverse relationship between IC and ADL (Pearson’s *r* − 0.12) and IC and IADL (Pearson’s *r* − 0.24) [17]. A higher IC is also associated with a lower incidence of falls, dependence, hospitalization, and mortality [28–30].

A single easily measured asset-based scale of IC is lacking. Most measurements of IC use the absence of depression as the proxy measure of psychological capacity. However in our view, all IC domains should be measured in the positive (assets), hence our usage of satisfaction with life rather than depression for the psychology domain. A further major shortcoming of IC measurements is the absence of a standardized measurement across studies, which prevents comparability and inhibits wide clinical application [31]. Calculations of the various scores are often based on statistical modeling, such as the earlier validated scores from ELSA [15, 16] and the China Health and Retirement Longitudinal Study (CHARLS) [32] which, although robust in construct, are not directly translatable into clinical practice.

It is proposed that scoring systems using a scale of 0–10 may be more directly translatable [18]. Our findings of an inverse relationship between DSIC and adverse outcomes and the effect sizes we found are in keeping with other studies using scales measuring IC on a 0–10 summative scale, where domains have designated values between 0 and 2 for each of the five domains [33–35]. These studies are feasibly performed prospectively in various healthcare settings including the community and in hospital. Going forward, IC should be measured using validated, clinically meaningful, easily calculated scales which require no additional training to deliver.

Although the direction of association was the same, the effect sizes for prediction of adverse outcomes found in this study using the same ELSA population (albeit with different waves) differed to that found by Campbell et al. [16]. One reason for this may be that the Campbell scale had a standardized mean of 50 and standard deviation of 10, whereas the DSIC had a range of 0–10. Hence, a 1-unit increase in the former scale was not directly comparable to a 1-unit increase in the latter. In addition, our study design excluded participants with incomplete intrinsic capacity data, whereas Campbell et al. used a full information maximum-likelihood technique to estimate missing values.

Pain is a very common problem in older people. In this dataset, 36% answered yes to the question ‘Are you often troubled with pain?’. This is comparable to the population prevalence for chronic pain of 40–55% [36] for people aged 65 and over. People with chronic pain have more difficulties in ADL and higher mortality, compared to those with no pain [37–39]. In addition, people with pain (vs no pain), and more severe pain (vs milder pain), were more likely to have lower IC [10, 11].

Our study adds to the understanding of this triangulated relationship by illustrating how changes in pain contribute to changes in IC and functional outcomes. We found that people with pain at baseline and no pain at follow-up had a flatter longitudinal IC trajectory compared to other groups, suggesting that improvement of pain contributed to maintenance of IC. In addition, the model we created for the interaction between pain, IC, and functional outcomes accounted well for ADL and IADL difficulties. ADL and IADLs are well intercorrelated [40], which likely explains the close values we found in both models. Further, multimorbidity, pain, and IC all had a direct effect on ADL difficulties of similar magnitude (StdBeta of 0.11, 0.13, and − 0.12 respectively). Therefore, improving pain and maintaining IC are at least as important, and much more targetable, than treating multimorbidity, in order to prevent ADL losses and maintain independence. This is consistent with the previous reports that achieving remission from pain helped to prevent functional declines in ADL [39], and with studies aiming to improve pain and optimize IC [41, 42] where the outcome was maintenance of functional abilities.

While age did have a direct effect on ADL difficulty per se, it did cause a decline in ADL functioning mediated by IC losses. Our findings suggest that advancing age in itself does not impair ADL, but rather acts through declining IC to contribute to these disabilities. Age was also not an independent determinant in the development of ADL difficulties in a German cohort study [40]. Rather, social, environmental, and physical (e.g., cognitive, nutritional, functional) factors determined whether someone developed ADL difficulties [40, 43].

### Strengths and limitations

Strengths of this study include its longitudinal design, the description of pain trajectories in the context of IC and adverse outcomes, and comprehensive adjustment for potential confounding factors. In addition, we have created and validated an asset-based user-friendly scale of intrinsic capacity that is ready to use in any clinical setting.

This study had some limitations that should be acknowledged. Owing to missing IC and outcomes data in wave 6, only 61% (*n* = 2486) of the original cohort from wave 4 could be examined for associations between pain, IC and outcomes. It may be that some of the missing IC and outcomes values in wave 6 were due to people who were unwell or who had died, hence overlooking the number of difficulties in each outcome. The number of events in some outcomes categories (e.g., need for formal help) was small, and so, inferences about associations and causality should be made with caution.

The trajectories of pain were based on whether people were troubled with pain during their interview for both waves 4 and 6. This does not take into account the changing and dynamic nature of pain over the course of the intervening 4 years between these waves. The categorization of pain based on self-report can introduce recall bias, leading to pain over- or underestimation. The dichotomized nature of whether or not one has pain is rather blunt, but nevertheless fulfills our research question of enabling a broad overview of how pain interacts in the context of IC and dependence, daily living abilities, and falls. Future research should consider more refined exploration of how pain intensities, sites, and causes influence IC and management strategies to improve the same.

## Conclusion

The DSIC is a user-friendly tool to measure intrinsic capacity (IC) and has construct and predictive validity in people aged 60 and over. People with pain at baseline and follow-up had lower IC compared to people with no pain at any point in their trajectory. Pain was associated with poorer IC and mediated the relationship between IC and ADL difficulties.

## Supplementary Information

Below is the link to the electronic supplementary material.

Supplementary file1 (DOCX 1795 KB)

## Data Availability

The data underlying this article are available in the UK Data Archive at https://ukdataservice.ac.uk/ and can be accessed with SN 5050.
