# Supplementary material for: Validity of the Derby Scale of Intrinsic Capacity and its association with Pain: a usable measurement of intrinsic capacity based on the English Longitudinal Study of Ageing
Source: Eur Geriatr Med. 2026 Jul 23;17(5):2499–507. doi: 10.1007/s41999-026-01558-z (PMC13754766; doi:10.1007/s41999-026-01558-z)
Supplement: Supplementary file 1 [file 41999_2026_1558_MOESM1_ESM.docx]

**Supplementary Files**

*Supplementary Figure 1: Relationship between the Derby Scale of Intrinsic Capacity and the Factor Score Sum from Campbell et al, 2022.*

*Supplementary Table 1: The Derby Scale of Intrinsic Capacity (DSIC) scoring system. *Vision is corrected with refractive lens, if appropriate and Hearing is corrected with hearing aids, if appropriate*.

|  |  | **Score** | | | | |
| --- | --- | --- | --- | --- | --- | --- |
| **Domains** | **Items** | **0** | **0.5** | **1** | **2** | **Total** |
| **Locomotion** | **Short Physical Performance Battery** | 0-6 | - | 7-9 | 10-12 | 2 |
| **Vitality** | **Body Mass Index** | <18.5 or >=30 | - | 25-29.9 | 18.5-24.9 | 2 |
| **Sensory a)** | **Self-reported Vision*** | Poor or registered blind | Fair or Good | Very Good or Excellent | - | 1 |
| **Sensory b)** | **Self-reported Hearing*** | Poor | Fair or Good | Very Good or Excellent | - | 1 |
| **Psychology** | **Satisfaction with Life Scale** | 0-19 | - | 20-24 | 25-35 | 2 |
| **Cognition** | **Orientation 4 questions (day of the week, day of the month, month, year) and 10 word immediate recall** | Orientation <4 correct and 10 word recall 0-4 words | - | Orientation 4 correct and 10 word recall 0-4 words  *Or*  Orientation <4 correct and 10 word recall 5 or more words | Orientation 4 correct and 10 word recall 5 or more words | 2 |

*Supplementary Figure 2: Distribution of DSIC scores in the cohort from Wave 4.*

*Supplementary Table 2: Covariates No Pain vs Pain baseline*

|  | No Pain | Pain | OR (95% CI) | p-value |
| --- | --- | --- | --- | --- |
| Sex  Male (ref)  Female, n | 1317  1292 | 614  829 | 1.376 (1.209, 1.567) | <0.001 |
| Age*  Mean (SD) |  |  | 1.00 (0.99, 1.01) |  |
| Marital status  Married or in civil partnership (ref)  Separated, divorced, or never married | 1881  728 | 1028  415 | 1.043 (0.905, 1.203) | 0.562 |
| Employment (1 missing)  Employed (ref)  Semi retired or retired  Unable to work  Not working | 557  1891  14  147 | 259  1035  37  111 | 1.18 (0.99, 1.39)  5.68 (3.02, 10.70)  1.62 (1.22, 2.16) | 0.054  <0.001  <0.001 |
| Smoking (374 missing)  No (ref)  Yes | 2135  234 | 1170  139 | 1.084 (0.868, 1.353) | 0.494 |
| Alcohol (23 missing)  No (ref)  1-4 days drinking in past week  5 or more days drinking in past week | 766  1147  680 | 497  614  325 | 0.825 (0.710, 0.958)  0.737 (0.619, 0.876) | 0.012  <0.001 |
| Education (22 missing)  Degree (ref)  Higher educational non-degree  A level or equivalent  O level or equivalent  No qualification | 509  439  187  849  611 | 198  213  104  477  443 | 1.247 (0.989, 1.573)  1.430 (1.069, 1.912)  1.444 (1.184, 1.762)  1.864 (1.519, 2.287) | 0.062  0.016  <0.001  <0.001 |
| Number of conditions (41 missing)  No long term conditions (ref)  1 or 2 long term conditions  3 or more long term conditions | 1121  1152  304 | 457  598  379 | 1.273 (1.099, 1.475)  3.058 (2.539, 3.683) | 0.001  <0.001 |
| Physical activity levels (3 missing)  High (ref)  Moderate  Low  Sedentary | 663  1468  419  57 | 233  758  396  55 | 1.469 (1.236, 1.747)  2.689 (2.196, 3.294)  2.746 (1.842, 4.094) | <0.001  <0.001  <0.001 |

**Exp(B) reported from linear regression analysis. Binary logistic regression used in all except for age.*

*Supplementary Table 3: Groups of trajectories of pain and associations with health and care outcomes*

|  | **Pain 🡪 Pain**  **N=533** | **Pain 🡪 No Pain**  **N=287** | **No Pain 🡪 Pain**  **N=380** | **No Pain 🡪 No Pain, n=1286** |
| --- | --- | --- | --- | --- |
| **Falls**  **Yes**  **OR (95% CI)** | 34% | 25.1% | 23.4% | 21.7% |
|  | 0.88 (0.77, 1.01) | 0.90 (0.74, 1.09) | 0.85 (0.72, 1.01) | 0.99 (0.89, 1.11) |
| **Any difficulty with ADL or IADL, Yes**  **OR (95% CI)** | 42.2% | 19.9% | 23.4% | 9.0% |
|  | 0.734 (0.642, 0.839) | 0.77 (0.62, 0.95) | 0.78 (0.66, 0.93) | 0.69 (0.59, 0.79) |
| **Informal help**  **Yes**  **OR (95% CI)** | 20.3% | 9.1% | 10.3% | 4.0% |
|  | 0.72 (0.62, 0.85) | 0.79 (0.59, 1.06) | 0.68 (0.54, 0.85) | 0.69 (0.56, 0.85) |
| **Formal help**  **Yes**  **OR (95% CI)** | 5.3% | 2.8% | 2.1% | 1.0% |
|  | 0.66 (0.50, 0.87) | 0.59 (0.35, 1.00) | 0.55 (0.35, 0.85) | 0.60 (0.40, 0.89) |

*Unadjusted Binary Logistic Regression of independent variable (baseline IC) and dependent variable (health and care outcomes), according to pain groups and the proportion of adverse outcome per group. Exp(B) reported as Odds Ratios.*

*Supplementary Table 4: Structural Equation Modelling values for Activities of Daily Living (ADL) difficulties.*

|  | **Beta** | **SE** | **p-value** | **Standardised Beta** |
| --- | --- | --- | --- | --- |
| Number of ADL difficulties | | | | |
| DSIC at wave 6 | -0.07 | 0.02 | <0.001 | -0.123 |
| Log Pain Severity | 0.485 | 0.129 | <0.001 | 0.132 |
| Log Age | 0.597 | 0.59 | 0.312 | 0.036 |
| Number of Comorbidities | 0.051 | 0.016 | 0.001 | 0.112 |
| DSIC at wave 6 | | | | |
| Log Pain Severity | -0.955 | 0.283 | 0.001 | -0.118 |
| Log Age | -6.494 | 1.279 | <0.001 | -0.178 |
| Number of Comorbidities | -0.141 | 0.034 | <0.001 | -0.146 |
| Log Pain Severity | | | | |
| Log Age | 0.028 | 0.159 | 0.859 | 0.006 |
| Number of Comorbidities | 0.013 | 0.004 | 0.002 | 0.114 |

*Supplementary Figure 3: Structural Equation Modelling of the effects of age and number of comorbidities on Instrumental Activities of Daily Living (IADL) difficulties, moderated by pain and intrinsic capacity.*

*Figures stated on arrows are Standardised Beta. Only significant Standardised Beta values are shown, all p<0.01. Green arrows indicate a positive relationship, and orange arrows indicate an inverse relationship.*
